# Influence of predictors on postoperative quality of life in women with breast neoplasms

**DOI:** 10.1590/0034-7167-2025-0245

**Published:** 2026-10-02

**Authors:** Michele Cunha Silva, Juliana Maria de Paula Avelar, Mariana Torrglosa Ruiz, Adriana Cristina Nicolussi

**Affiliations:** I Universidade Federal do Triângulo Mineiro. Uberaba, Minas Gerais, Brazil; II Centro Universitário Estácio de Ribeirão Preto. Ribeirão Preto, São Paulo, Brazil

**Keywords:** Breast Neoplasms, Breast Cancer Lymphedema, Surgical Oncology, Quality of Life, Postoperative Period., Neoplasias de la Mama, Linfedema del Cáncer de Mama, Oncología Quirúrgica, Calidad de Vida, Período Postoperatorio.

## Abstract

**Objectives:**

to evaluate the presence and influence of lymphedema and sociodemographic and clinical variables on the health related quality of life of women with breast cancer who underwent surgery.

**Methods:**

a prospective cohort study was conducted with 90 women with breast cancer. Sociodemographic and clinical questionnaires were used, along with the QualityofLife Questionnaire-Core30 and BreastCancer26. Descriptive analysis, association tests, and multiple linear regression were performed.

**Results:**

lymphedema occurred in 37 women. A reduction in role functioning and an increase in symptoms in the affected arm were observed. Positive improvements were detected with reductions in insomnia, constipation, distress related to hair loss and increases in future perspectives. In the associations, women with children reported more insomnia, and those who underwent mastectomy showed better global health status and future perspectives.

**Conclusions:**

having children was a predictor of insomnia, and the type of surgery influenced future perspectives and overall quality of life in these women.

## RESUMEN

**Objetivos:**

evaluar la presencia e influencia del linfedema y de variables sociodemográficas y clínicas sobre la calidad de vida relacionada con la salud de mujeres con cáncer de mama sometidas a cirugía.

**Métodos:**

se realizó un estudio de cohorte prospectivo con 90 mujeres. Se utilizaron cuestionarios sociodemográfico y clínico, *QualityofLife Questionnaire-Core30* y *BreastCancer26*. Se llevaron a cabo análisis descriptivos, pruebas de asociación y regresión lineal múltiple.

**Resultados:**

el linfedema se presentó en 37 mujeres. Se observó una reducción en el desempeño de roles y un aumento de síntomas en el brazo afectado. Se detectaron mejoras con la reducción del insomnio, del estreñimiento, de la molestia por la pérdida de cabello y con el aumento de las perspectivas futuras. En las asociaciones, las mujeres con hijos reportaron más insomnio, y aquellas sometidas a mastectomía presentaron mejor estado global de salud y mejores perspectivas futuras.

**Conclusiones:**

tener hijos fue un predictor de insomnio, y el tipo de cirugía influyó en las perspectivas futuras y en la calidad de vida general de estas mujeres.

## INTRODUCTION

Breast cancer is the type of neoplasm that most affects women worldwide. In Brazil, 74,000 new cases are expected between 2023 and 2025, corresponding to an estimated risk of 66.54 new cases per 100,000 women^(1)^.

Several factors have influenced the health related quality of life (HRQoL) of women with breast cancer. This neoplasm is becoming increasingly common among women of reproductive age. A study on the attitudes and experiences of breast cancer survivors revealed that 76% of young patients with cancer who do not have children wish to conceive, and 35% of those who already have children hope to have another in the future^(2)^. This concern about being able to procreate after treatment has triggered psychological distress and worry among these women.

A social factor that affects health is education. One study showed that low levels of schooling negatively influence the ability to understand instructions at the time of diagnosis and treatment. On the other hand, higher levels of education positively motivate the prompt completion of examinations as well as faster initiation of treatment^(3)^. Additionally, women with lower educational levels are more likely to be diagnosed at an advanced stage of breast cancer^(4)^.

For the selection of breast cancer treatment, individualized characteristics are considered, such as tumor staging, genetic factors, genomic signatures, and patient preferences for personalized care. Treatment may include a combination of chemotherapy, radiotherapy, surgery, endocrine therapy, and immunotherapy, and requires continuous follow up and reassessment according to patients’ experiences and treatment responses^(5,6)^.

Surgery is considered the main treatment for breast cancer, and the type of surgery performed is another factor that may influence women’s HRQoL. Surgical interventions include conservative procedures (quadrantectomy and lumpectomy) and radical procedures (mastectomy), with or without axillary lymphadenectomy^(6)^, which may lead to the development of lymphedema. Furthermore, age over 45 years is a significant factor for the development of lymphedema in modified radical mastectomies^(7)^.

Lymphedema is characterized by a high concentration of protein rich fluid within the interstitial space caused by a total or partial obstruction of lymphatic drainage, resulting in tissue edema^(8)^. Breast cancer-related lymphedema may occur as a postoperative complication caused by lymph leakage into the interstitium and once developed, it compromises women’s psychosocial, emotional, and functional capacities, directly affecting their HRQoL^(7,9)^.

Lymphedema may cause pain, cyanotic skin, swelling, paresthesia, decreased or lost shoulder range of motion, and changes in upper limb circumference. Its onset may occur immediately after surgery or appear later, even years after the procedure^(7)^, with an incidence of approximately 20% to 30% of breast cancer cases and a prevalence rate of 15% to 30%^(10)^.

Despite significant advances in breast cancer therapies, the HRQoL of women undergoing treatment is still negatively affected. They experience emotional and psychological distress, functional impairment, pain, and fatigue^(11)^. Therefore, the complexity of treatment and the entire oncological context intensify women’s biopsychosocial and spiritual needs, making comprehensive and individually tailored interventions necessary^(12)^.

Given the above, and in order to clarify the influence of factors on the health related quality of life of women who undergo breast surgery, this study was proposed with the aim of supporting future interventions by the healthcare team that assists them and improving these women’s HRQoL.

## OBJECTIVES

To evaluate the presence and influence of lymphedema and sociodemographic and clinical variables on the health related quality of life of women with breast cancer who have undergone surgery.

## METHODS

### Ethical aspects

The study was approved by the Research Ethics Committees (REC) of the proposing institution (CAAE 52218021.0.0000.5154) and the collaborating institution (CAAE 52218021.0.3003.0149), and the Free and Informed Consent Form was obtained from all participants in two copies.

### Setting, study design and period

This is a prospective cohort study guided by the STROBE tool. It was conducted between March 2022 and May 2023 in two public hospitals that are reference centers for breast cancer treatment in the city of Uberaba, in the southern region of *Triângulo Mineiro*, Brazil.

### Sample, inclusion and exclusion criteria

For the sample size calculation, an a priori coefficient of determination (R^2^ = 0.13) was considered in a linear regression model with five predictors, with a significance level or type I error of α = 0.05 and a type II error of β = 0.1, resulting in an a priori statistical power of 80%. Considering a 20% sample loss and a maximum recruitment attempt of 163 individuals, a sample size of 94 participants was obtained.

A non probabilistic convenience sampling method was used, and women aged 18 years or older, diagnosed with breast cancer, and undergoing surgery were included. A total of 114 women were approached in the preoperative period, of whom one refused to participate and 23 were excluded due to loss to follow up - therefore, the final sample of this study consisted of 90 women.

### Study Proctocol

For this study, a questionnaire was used containing the following sociodemographic and clinical variables: age, self reported race, marital status, whether the participant had children, occupation, educational level, affected breast, family history of breast cancer, type of surgery, and other treatments performed, such as chemotherapy and radiotherapy.

To determine the presence of lymphedema, upper limb perimetry was performed according to pre established criteria^(13)^. For measurement, the patient must be seated with her arms hanging alongside the trunk and the chest uncovered. The limb is measured at 7 cm and 14 cm above the elbow joint line, and at 7 cm, 14 cm, and 21 cm (wrist) below the elbow joint line. Lymphedema is classified as mild (less than 3 cm), moderate (3 to 5 cm), and severe (greater than 5 cm)^(7)^.

To assess health related quality of life (HRQoL), the Quality of Life Questionnaire Core 30 (QLQ C30) and the Quality of Life Questionnaire Breast Cancer 23 (QLQ BR23) were administered. The QLQ C30 consists of 30 questions comprising five functional scales-physical, emotional, cognitive, social, and role functioning; one Global Health Status/Quality of Life (GHS/QoL) scale; three symptom scales-fatigue, pain, nausea and vomiting; five single items assessing symptoms such as dyspnea, insomnia, loss of appetite, constipation, and diarrhea; and one item assessing financial difficulties. The instrument has been translated into Portuguese and validated in Brazil^(14)^.

The QLQ BR23 also has a translated and validated version for Brazilian Portuguese and is a specific module composed of 23 items related to breast cancer. It includes five multi item scales that assess treatment side effects (surgery, chemotherapy, radiotherapy, hormone therapy), arm and breast symptoms, body image, and sexual functioning. It also contains single items that assess sexual satisfaction, distress related to hair loss, and future perspectives^(15)^.

For both HRQoL instruments, scores are calculated and transformed into a scale from 0 to 100. For the functional scales and the GHS/QoL scale, values closer to 100 are considered better, whereas for symptom scales and items, higher values indicate a greater presence of symptoms^(14,15)^.

Data collection was carried out by a physiotherapist and graduate student, along with three undergraduate nursing students, all previously trained by the lead researcher (advisor). Based on the weekly surgical schedule, patients who met the inclusion criteria were approached in the immediate preoperative period - considered Time 1 (T1) - and invited to participate. After providing consent, participants completed the sociodemographic and clinical questionnaire and the HRQoL instruments individually through interviews, in addition to undergoing upper limb circumference measurements, which were recorded on printed forms.

Patients were approached again at a second time point, Time 2 (T2), approximately 45 days after surgery (with a variation of about three days due to weekends and holidays) during an outpatient postoperative consultation, to complete the HRQoL instruments again and undergo upper limb perimetry.

### Statistics and analysis of the results

The data were coded and entered into an Excel spreadsheet and validated after double entry. For the statistical analysis, the Statistical Package for Social Science for Windows (SPSS), version 29.0.1.0, was used.

Categorical variables were analyzed using descriptive statistics with absolute and relative frequency distributions, while for the HRQoL instruments, scores were calculated followed by measures of central tendency (mean) and variability (standard deviation). The data were tested for distribution, and to assess differences in HRQoL scores before and after surgery, a paired t test was performed.

To evaluate the influence of sociodemographic and clinical variables on HRQoL, Student’s t test for independent groups was applied to five dichotomous predictors in relation to the HRQoL instruments, namely: age group (< 60 and ≥ 60 years), having children (yes or no), education level (no schooling to high school vs. higher education to postgraduate), type of surgery (mastectomy vs. other types), and lymphedema (presence or absence). Pearson correlations were used for age in complete years, and Spearman correlations for education level across categories.

A multiple linear regression analysis was also conducted to examine the simultaneous influence of the five predictors (lymphedema, having children, type of surgery, education level, and age) on the primary outcome: the Global Health Status (GHS/QoL) score. The study adopted a statistical significance level of 5% (α = 0.05)

## RESULTS

A total of 90 women participated, with a mean age of 55 years (SD = 11.91) and ages ranging from 18 to 82 years. Most women self identified as white (60.0%), were married or in a stable union (47.8%), and had children (86.7%). There was a similar distribution between those who were economically active and those who were homemakers, retirees, or pensioners. Regarding educational level, most had completed elementary school, followed by high school and higher education. Two women reported never having attended school, and one had completed postgraduate studies, as shown in Table 1.

**Table 1:** Sociodemographic characterization of the sample (N=90), Uberaba, Minas Gerais, Brazil, 2022-2023

Variables	Characteristics	Total n (%)
Self-reported color	White *Mulata* Black Yellow	54 (60.0) 18 (20.0) 17 (18.9) 1 (1.1)
Marital Status	Married / Stable Union Single Divorced Widow	43 (47.8) 20 (22.2) 16 (17.8) 11 (12.2)
Has sons/daughters?	Yes No	78 (86.7) 12 (13.3)
Profession / Occupation	Economically active Homemakers Retired/Pensioners Not employed	44 (48.9) 24 (26.7) 20 (22.2) 2 (2.2)
Level of Education	No schooling Incomplete elementary education Complete elementary education Incomplete high school education Complete high school education Incomplete higher education Complete higher education Completed postgraduate education	2 (2.2) 25 (27.8) 22 (24.4) 6 (6.7) 19 (21.1) 1 (1.1) 14 (15.6) 1 (1.1)

Table 2 shows that there was a small difference between the affected breasts, with the right side being slightly more prevalent (50.0%). Most women reported having no family history of breast cancer (56.7%), underwent breast conserving surgery (66.7%), and did not receive chemotherapy (51.1%) or radiotherapy (83.3%).

**Table 2:** Clinical and therapeutical characterization of the sample (N=90), Uberaba, Minas Gerais, Brazil, 2022-2023

Variables	Characteristics	Total n (%)
Affected breast	Right Left Both	45 (50.0) 43 (47.8) 2 (2.2)
Family history	No Yes	51 (56.7) 38 (42.3)
Type of surgery performed	Conservative Mastectomies	60 (66.7) 30 (33.3)
Underwent chemotherapy	No Yes	46 (51.1) 43 (47.9)
Underwent radiotherapy	No Yes	75 (83.3) 14 (15.7)

Lymphedema in the limb ipsilateral to the affected breast occurred in 37 women (41.1%) in the postoperative period, in at least one of the measured sites. The highest incidence of lymphedema was observed 14 cm above the elbow, as shown in Table 3. The degree of lymphedema presented by the women was classified as moderate (3 to 5 cm difference).

**Table 3:** Distribution of lymphedema in the limb ipsilateral to the operated breast in the sample (N=90), Uberaba, Minas Gerais, Brazil, 2022-2023

Measurement of the limb ipsilateral to the operated breast	Lymphedema present n (%)	Lymphedema absent n (%)
14 cm above elbow	19 (21.1)	71 (78.9)
7 cm above elbow	9 (10.0)	81 (90.0)
7cm below elbow	14 (15.6)	76 (84.4)
14 cm below elbow	5 (5.6)	85 (94.4)
21 cm below elbow	13 (14.4)	77 (85.6)

Table 4 presents the results of the QLQ C30 and QLQ BR23. Global Health Status and the physical, role, cognitive, and social functioning scales showed a slight reduction from the pre to the postoperative period, with statistical significance observed only for role functioning (p < 0.05), while emotional functioning remained unchanged.

**Table 4:** Mean, standard deviation, and paired t test for the scales and items of the QLQ C30 and QLQ BR23 instruments (N=90), Uberaba, Minas Gerais, Brazil, 2022-2023

Scales and Itens	Mean (SD) (preoperative)	Mean (SD) (postoperative)	*p* value
Global health status/Quality of life	82.77 (19.18)	81.94 (19.99)	0.74
Physical functioning	90.81 (11.97)	88.44 (15.21)	0.14
Role functioning	89.81 (20.85)	84.07 (21.91)	0.04^*^
Cognitive functioning	75.18 (23.60)	74.44 (29.58)	0.81
Social functioning Emotional functioning	88.14 (21.23) 75.18 (23.80)	84.62 (23.33) 75.55 (26.04)	0.21 0.88
Fatigue	12.46 (17.69)	15.67 (19.41)	0.13
Nausea and vomiting	8.14 (18.39)	6.85 (15.37)	0.49
Pain	11.84 (16.42)	15.92 (22.47)	0.09
Dyspnea	10.36 (20.98)	7.03 (20.89)	0.18
Insomnia	46.66 (44.07)	33.33 (40.59)	0.00^*^
Loss of appetite	14.07 (25.95)	12.59 (24.77)	0.62
Constipation	9.99 (21.46)	4.44 (15.95)	0.02^*^
Diarrhea	7.77 (21.80)	7.77 (19.38)	1.00
Financial difficulties Body image Sexual functioning Sexual satisfaction Future perspectives Side effects related to the treatment Breast symptoms Arm symptoms Upset by hair loss	19.62 (28.21) 88.23 (20.22) 9.99 (19.31) 10.73 (22.80) 59.25 (38.95) 23.82 (17.85) 11.47 (15.12) 15.98 (23.64) 20.74 (35.18)	18.88 (30.00) 86.46 (22.50) 14.81 (21.70) 14.81 (23.49) 71.47 (32.59) 23.50 (20.43) 15.55 (18.36) 23.01 (25.91) 12.59 (27.63)	0.82 0.42 0.08 0.21 0.00^*^ 0.87 0.10 0.02^*^ 0.03^*^

* *Significant difference at the 5% level (p < 0.05).*

Regarding symptoms, the main complaint was insomnia, which-along with constipation-showed a statistically significant reduction from pre to postoperative assessment, whereas fatigue and pain increased postoperatively.

In the QLQ BR23 evaluation, women reported a decrease in body image and an increase in breast and arm symptoms, with the latter reaching statistical significance. Despite this increase in complaints, they also reported improvements in sexual functioning and satisfaction, better future perspectives, and reduced distress related to hair loss, with the last two showing statistically significant differences, as shown in Table 4

In the analysis of predictors of HRQoL, statistically significant differences were observed for the symptom insomnia (p = 0.04), in which women who had children (78-70.2%) reported experiencing more insomnia (M = 36.75, SD = 41.11) than those without children (M = 11.11, SD = 29.58). Women who underwent mastectomy (30-27.0%) showed better GHS/QoL (p = 0.01, M = 89.44, SD = 12.56) and better future perspectives (FP) (p = 0.04, M = 81.10, SD = 20.87) compared with those who underwent other types of surgery (GHS/QoL: M = 78.19, SD = 21.97; FP: M = 66.66, SD = 36.30).

Regarding the simultaneous influence of the five predictors (lymphedema, having children, type of surgery, education level, and age) on the primary outcome (Global Health Status score), the multiple linear regression model indicated that the type of surgery performed was the only variable that significantly predicted higher GHS scores in these women (p = 0.01), as shown in Table 5.

**Table 5:** Multiple linear regression analysis of predictors and Global Health Status/Quality of Life in the postoperative period (N=90), Uberaba, Minas Gerais, Brazil, 2022-2023

General Health Situation	β	*p* value
Lymphedema	-0.06	0.55
Sons / Daughters	-0.09	0.41
Surgery	0.26	0.01^*^
Level of Education	-0.01	0.90
Age	0.09	0.42

* *Significant difference at the 5% level (p < 0.05).*

Pearson’s correlation was applied to the variable age in complete years (p = 0.42), and Spearman’s correlation was applied to the variable education level in its different categories (p = 0.90); however, neither showed statistically significant results

## DISCUSSION

The profile of the women in this study was consistent with research conducted in Franca, São Paulo, which found a mean age of 59.9 years and a majority who self identified as white (78.1%). However, it differed regarding the type of surgery, with mastectomies predominating in 55.6% of patients^(16)^. In contrast, a study conducted in Nigeria reported a lower mean age of 45.94 years (SD = 9.08); most women were married (58.3%) and had one to four children, and the surgical profile also diverged, with mastectomy (83.3%) far exceeding lumpectomy (16.7%)^(17)^.

Regarding lymphedema, in the present investigation it occurred in 41.1% of the women. In a study conducted in São Paulo, lymphedema was detected in 47.5% of women; among them, 57.1% had undergone conservative surgery and 42.9% radical surgery. The authors also found a statistically significant association (p = 0.06) with postoperative time, with an increased likelihood of lymphedema occurring after six months post surgery^(7)^. An increase in lymphedema rates of 10% and 18% was also found at 12 and 18 months postoperatively, respectively, in the United States^(18)^.

With respect to HRQoL, a slight decline in the evaluated functions was observed from pre to postoperative periods, corroborating a study conducted in the United States in which physical, psychological, and social domains decreased during the first month after surgery but improved approximately six months later^(18)^. In Saudi Arabia, psychological, social, and emotional challenges significantly affected overall well being and HRQoL^(19)^. Meanwhile, in Nigeria, psychological distress was significantly associated with QoL (p = 0.001), as women reporting psychological distress had lower QoL (M = 68.42, SD = 16.22) compared with those without distress (M = 85.52, SD = 15.99)^(17)^.

Among the symptoms, insomnia stands out. Although it decreased in the postoperative period, it remained the most frequently reported symptom at both time points and was significantly more common among women with children. Insomnia is considered a common symptom and is related to several factors to which women are exposed during treatment, such as pain and local discomfort, treatment side effects, and emotional distress^(20)^.

The present investigation found reports of decreased body image, which - although not statistically significant - corroborates an integrative review showing that women who undergo mastectomy face major challenges due to breast removal, which affects their self perception and self esteem and compromises their sexuality^(21)^.

Breast surgery caused conflicts in intimate relationships among younger women (between 18 and 40 years old), as many felt ashamed to be naked in front of their partners when resuming sexual activity, perceiving themselves as having a “defective body”, which reminded them of their illness^(22)^.

Changes in physical appearance caused by mastectomy and hair loss led to body image deficits in these young women. They reported that their sexual activity changed after diagnosis and treatment, and that resuming it was only possible with social and emotional support, especially from their partners^(22)^.

In this study, emotional function scores did not change from the pre to the postoperative period. However, another study showed that being younger (p = 0.005) and being referred for lymphedema treatment (p = 0.006) were associated with worse HRQoL in the psychological domain^(18)^.

A Lebanese study that used the same HRQoL instruments to assess women before and one day after breast conserving surgery found a statistically and clinically significant reduction in body image, as well as in physical and emotional functioning in the postoperative period. It also reported a positive change in physical functioning among married women. Reduced body image and future perspectives were identified as risk factors for poor quality of life^(23)^, which aligns with the present investigation, in which women who underwent conservative surgery had lower scores for future perspectives and quality of life than those who underwent mastectomy.

However, the results differ from another study that also compared outcomes with the option of breast reconstruction surgery. That study found that women who underwent oncoplastic surgery had better QoL than those who had conservative surgery, and that women who had conservative surgery had better QoL than those who underwent mastectomy without reconstruction^(24)^.

Among Egyptian women who underwent mastectomy, QoL was influenced by several factors: they had a global QoL score of 42.0, and the most impaired scales were role functioning and emotional functioning. The most prevalent symptoms were fatigue (65%), insomnia (61%), and pain (60%)^(25)^.

Given the various changes that may occur as a result of surgeries for breast cancer treatment, healthcare professionals should propose interventions that can help these women reduce physical and psychosocial symptoms and improve functioning and HRQoL.

Although the present investigation did not find a statistically significant association between lymphedema and quality of life, this postoperative complication should still be assessed and treated. In a study that used an adapted form of complex decongestive therapy (CDT) in a patient to control symptoms and lymphedema volume, physiotherapists provided guidance on skin care, home passive exercises, and compressive bandaging twice a week. Even without lymphatic drainage, there was a reduction in lymphedema volume. In the subsequent maintenance phase, a compression sleeve was used daily. The treatment also reduced pain and the sensation of heaviness in the limb, as reported by the patient, whose adherence to the proposed therapy contributed to the positive outcomes^(26)^.

To improve symptoms, including insomnia, psychosocial^(17)^ and behavioral therapies are recommended, such as cognitive behavioral therapy, yoga, mind-body practices^(20)^, kinesiotherapy^(27)^, as well as guidance on physical activity, balanced nutrition, and maintaining healthy lifestyle habits^(28)^. Sleep hygiene practices should also be encouraged, along with the provision of social support - such as emotional, informational, and instrumental assistance - to women with children, offered by the healthcare professionals who care for this population. Nurses, along with other professionals such as physiotherapists, psychologists, and physical educators, should propose actions aimed at improving HRQoL in women undergoing breast cancer treatment.

An example of such an intervention is the practice of physical activity, which is considered safe and can be performed throughout the entire treatment, provided that the woman is evaluated and monitored, including in the postoperative period. Physical activity results in reduced psychological symptoms - such as anxiety and depression - and other symptoms such as pain, fatigue, sleep disturbances, nausea, and vomiting, in addition to improving functional abilities and consequently enhancing well being and HRQoL^(28)^.

Another intervention used to improve quality of life is kinesiotherapy. When applied in the postoperative period of breast cancer, following a protocol that included 10 sessions of stretching, free active exercises, and resistance exercises administered by a physiotherapist, it was shown that patients who underwent the therapy experienced significant improvements in physical functioning (p = 0.01), role functioning (p = 0.02), fatigue (p = 0.03), pain (p = 0.01), insomnia (p = 0.02), and breast (p = 0.01) and arm symptoms (p = 0.01), along with a worsening of diarrhea (p = 0.02). Other scores assessed by the QLQ C30 and QLQ BR23 instruments also showed improvements, although without statistically significant differences^(27)^.

### Study limitations

As limitations, it is important to highlight the difficulty in contacting the women for postoperative data collection due to the anticipation or rescheduling of appointments. Another limitation was the absence of additional assessments over time. Although this is considered a limitation, it also represents an opportunity for future studies with the same population using different research designs

### Contributions to the field of Nursing, Health and Public Policies

The study contributes to the healthcare team that assists this population, as it provides information on possible conditions occurring in the pre and postoperative periods of these women. With this knowledge, nursing professionals, physiotherapists, and other healthcare providers can propose interventions to alleviate symptoms and dysfunctions that may affect patients’ HRQoL

## CONCLUSIONS

The presence of lymphedema occurred in less than half of the women evaluated. From the pre to the postoperative period, statistically significant changes (p < 0.05) were observed-negatively, with a reduction in role functioning and an increase in arm symptoms, and positively, with reductions in insomnia, constipation, and distress related to hair loss, as well as an increase in future perspectives.

Insomnia was the most prevalent symptom at both time points. As predictors, statistically significant associations were found indicating that women with children experienced more insomnia than those without, and that women who underwent mastectomy had better GHS/QoL and future perspectives scores than those who underwent conservative surgery.

Therefore, this study highlights the importance of healthcare professionals assessing the onset of lymphedema and other factors that may predispose women with breast cancer undergoing surgery to changes in quality of life. It also underscores the need to propose interventions that can alleviate or prevent treatment related symptoms, enhance biopsychosocial functioning, and consequently provide comprehensive care and better HRQoL for these women.

## Data Availability

The research data are available within the article.
