# Supplementary material for: Influence of predictors on postoperative quality of life in women with breast neoplasms
Source: Rev Bras Enferm. 2026 Oct 2;79:e20250245. doi: 10.1590/0034-7167-2025-0245 (PMC13631963; doi:10.1590/0034-7167-2025-0245)
Supplement: Translation

# Translation: Influência de preditores sobre a qualidade de vida pós-operatória de mulheres com neoplasias de mama

**DOI:** 10.1590/0034-7167-2025-0245pt

**Authors:** Michele Cunha Silva, Juliana Maria de Paula Avelar, Mariana Torrglosa Ruiz, Adriana Cristina Nicolussi

**Affiliations:** I Universidade Federal do Triângulo Mineiro. Uberaba, Minas Gerais, Brasil; II Centro Universitário Estácio de Ribeirão Preto. Ribeirão Preto, São Paulo, Brasil

**Keywords:** Neoplasias da Mama, Linfedema Relacionado a Câncer de Mama, Oncologia Cirúrgica, Qualidade de Vida, Período Pós-Operatório.

## Abstract

**Objetivos:**

avaliar a presença e influência do linfedema e de variáveis sociodemográficas e clínicas sobre a qualidade de vida relacionada à saúde de mulheres com câncer de mama submetidas à cirurgia.

**Métodos:**

estudo de coorte prospectivo realizado com 90 mulheres com câncer de mama. Utilizaram-se questionários sociodemográfico e clínico, *Quality of Life Questionnaire-Core30* e *BreastCancer26*. Realizada análise descritiva, testes de associações e regressão linear múltipla.

**Resultados:**

o linfedema ocorreu em 37 mulheres. Observou-se uma redução no desempenho de papel e aumento de sintomas no braço afetado. Melhoras positivas foram detectadas com redução da insônia, da constipação, perturbação com perda do cabelo e aumento nas perspectivas futuras. Nas associações, mulheres com filhos relataram mais insônia, e as mastectomizadas apresentaram melhor estado global de saúde e perspectivas futuras.

**Conclusões:**

ter filhos foi preditor para insônia e o tipo de cirurgia influenciou nas perspectivas futuras e na qualidade de vida geral destas mulheres.

## INTRODUÇÃO

O câncer de mama é o tipo de neoplasia que mais afeta as mulheres em todo o mundo. No Brasil, são esperados 74 mil casos novos entre os anos de 2023 a 2025, correspondendo a um risco estimado de 66,54 casos novos a cada 100 mil mulheres^(1)^.

Alguns fatores têm influenciado a qualidade de vida relacionada à saúde (QVRS) de mulheres com câncer de mama. Esta neoplasia está se tornando mais comum em mulheres em idade reprodutiva. Uma pesquisa sobre as atitudes e experiências das sobreviventes de câncer de mama revelou que 76% das pacientes jovens com câncer e sem filhos desejam conceber, e 35% das que já têm almejam ter outro filho no futuro^(2)^, e esta apreensão em poder procriar após o tratamento tem desencadeado preocupações e sofrimento psicológico nestas mulheres.

Um fator social que interfere na saúde é a educação. Um estudo mostrou que baixos níveis de escolaridade influenciam negativamente na capacidade de compreender instruções no momento do diagnóstico e tratamento. Por outro lado, níveis altos de escolaridade motivam positivamente a rápida realização de exames bem como a agilidade para iniciar o tratamento^(3)^. Além disso, mulheres com menor escolaridade apresentam maior probabilidade de serem diagnosticadas em estágio avançado para o câncer de mama^(4)^.

Para a escolha do tratamento do câncer de mama são consideradas características individualizadas, tais como: estadiamento do tumor, fatores genéticos, assinaturas genômicas e preferências das pacientes para um cuidado personalizado, e inclui combinação de quimioterapia, radioterapia, cirurgia, terapia endócrina e imunoterapia, devendo ter acompanhamento e reavaliações constantes de acordo com as experiências das pacientes e respostas ao tratamento^(5,6)^.

A cirurgia é considerada o principal tratamento para o câncer de mama e o tipo de cirurgia realizada é outro fator que pode influenciar a QVRS das mulheres. Dentre as intervenções cirúrgicas existem as conservadoras (quadrantectomia e tumorectomia) e as radicais (mastectomia) com associação ou não da linfadenectomia axilar^(6)^, que pode desencadear a formação de linfedema. Ademais, idade acima de 45 anos é significante para a formação de linfedema nas mastectomias radicais modificadas^(7)^.

O linfedema é caracterizado por uma alta concentração de fluídos com proteínas dentro do interstício ocasionado por uma obstrução total ou parcial da drenagem linfática, gerando edema tecidual^(8)^. O linfedema relacionado ao câncer de mama pode ocorrer como uma complicação do pós-operatório causado pelo extravasamento da linfa para o interstício e, uma vez adquirido, compromete as funções psicossocial, emocional e funcional da mulher, afetando diretamente a QVRS^(7,9)^.

O linfedema pode gerar dor, pele cianótica, edema, parestesia, diminuição ou perda da amplitude de movimento do ombro, e alterações na perimetria do membro superior. Seu início pode ocorrer logo no pós-operatório, ou tardiamente, surgindo anos após a cirurgia^(7)^, com incidência em torno de 20% a 30% dos casos de câncer de mama, e taxa de prevalência de 15% a 30%^(10)^.

Apesar do avanço positivo de terapias para o câncer de mama, a QVRS das mulheres em tratamento ainda é impactada negativamente. Elas apresentam sofrimentos emocionais e psicológicos, comprometimento funcional, dor e fadiga^(11)^. Portanto, a complexidade do tratamento e todo o contexto oncológico potencializa as necessidades biopsicossocioespirituais das mulheres, sendo necessárias intervenções abrangentes e adaptadas às características individuais^(12)^.

Diante do exposto, e a fim de elucidar a influência de fatores sobre a qualidade de vida relacionada à saúde das mulheres que são submetidas a cirurgias mamárias, foi proposto este estudo visando propor intervenções futuras pela equipe de saúde que as assiste e melhorar a QVRS destas mulheres.

## OBJETIVOS

Avaliar a presença e influência do linfedema e de variáveis sociodemográficas e clínicas sobre a qualidade de vida relacionada à saúde de mulheres com câncer de mama submetidas à cirurgia.

## MÉTODOS

### Aspectos éticos

A pesquisa foi aprovada pelos Comitês de Ética em Pesquisa (CEP) da instituição proponente (CAAE 52218021.0.0000.5154) e da instituicão coparticipante (CAAE 52218021.0.3003.0149), e o Termo de Consentimento Livre e Esclarecido foi obtido de todas as participantes em duas vias.

### Desenho, período e local do estudo

Trata-se de um estudo de coorte prospectivo, orientado pela ferramenta STROBE. Foi desenvolvido entre março de 2022 a maio de 2023 em dois hospitais públicos de referência em tratamento contra o câncer de mama na cidade de Uberaba, Triângulo Sul de Minas Gerais, Brasil.

### Amostra, critérios de inclusão e exclusão

Para o cálculo do tamanho amostral foi considerado um coeficiente de determinação apriorístico (R^2^=0,13) em um modelo de regressão linear com cinco preditores, com nível de significância ou erro do tipo I de α=0,05 e erro do tipo II de β=0,1, resultando em um poder estatístico apriorístico de 80%. Considerando uma perda de amostragem de 20% e o número máximo de tentativas de recrutamento de 163, obteve-se um tamanho de amostra de 94 participantes.

Foi realizada amostragem não probabilística por conveniência, sendo incluídas mulheres com 18 anos ou mais, diagnosticadas com câncer de mama, e submetidas à cirurgia. Foram abordadas 114 mulheres no pré-operatório, das quais uma se recusou e 23 foram excluídas por perda do seguimento - portanto a amostra final deste estudo contou com 90 mulheres.

### Protocolo do estudo

Para este estudo, utilizou-se um questionário contendo as seguintes variáveis sociodemográficas e clínicas: idade, cor autodeclarada, estado civil, se teve filhos, ocupação, nível de escolaridade, mama afetada, histórico familiar para câncer de mama, tipo de cirurgia e outros tratamentos realizados, como quimioterapia e radioterapia.

Para determinação da presença de linfedema, foi adotada a perimetria dos membros superiores (MMSS) de acordo com critérios pré-estabelecidos^(13)^, em que para mensuração a paciente deve estar sentada com os membros pendentes ao lado do tronco e com tórax despido. A medição do membro é feita a 7cm e 14cm acima da linha articular do cotovelo e 7 cm, 14cm e 21 cm (punho) abaixo da linha articular do cotovelo, sendo considerado a classificação do linfedema como leve (inferior a 3cm), moderado (3 a 5cm) e severo (acima de 5cm)^(7)^.

Para a avaliação da qualidade de vida relacionada à saúde (QVRS) foram aplicados os instrumentos *Quality of Life Questionnaire-Core30* (QLQ-C30) e *Quality of Life Questionnaire-Breast Cancer23* (QLQ-BR23). O QLQ-C30 é composto por 30 questões que compõem cinco escalas funcionais, sendo essas: funções física, emocional, cognitiva, social e desempenho de papel; uma de Estado Geral de Saúde/Qualidade de Vida (EGS/ QV); três escalas de sintomas: fadiga, dor, náusea e vômito; cinco itens para avaliar sintomas como: dispneia, insônia, perda de apetite, constipação e diarreia; e um item que avalia dificuldades financeiras, traduzido para o português e validado no Brasil^(14)^.

O QLQ-BR23 também possui versão traduzida e validada para o português brasileiro, sendo um módulo específico composto por 23 itens relacionados ao câncer de mama. Apresenta cinco escalas multi-item para avaliar os efeitos colaterais do tratamento (cirurgia, quimioterapia, radioterapia, terapia hormonal), sintomas relacionados ao braço e à mama, imagem corporal e função sexual. Também apresenta itens individuais para avaliar satisfação sexual, perturbação com a perda de cabelo e perspectivas futuras^(15)^.

Para ambos os instrumentos de QVRS os escores são calculados e transformados em uma escala de 0 a 100, sendo que, para as escalas funcionais e EGS/QV, os valores mais próximos de 100 são considerados melhores, enquanto para as escalas e itens de sintomas, valores maiores indicam presença de mais sintomas^(14,15)^.

A coleta de dados foi realizada por uma fisioterapeuta e estudante de pós-graduação e três alunas de graduação em enfermagem, previamente capacitadas pela pesquisadora responsável (orientadora). A partir da agenda cirúrgica semanal, as pacientes enquadradas nos critérios de inclusão eram abordadas no pré-operatório imediato, sendo este momento considerado Tempo 1 (T1), e convidadas à participação. Após consentimento delas, aplicava-se o questionário sociodemográfico e clínico e os instrumentos de QVRS de forma individual por meio de entrevistas, além da mensuração das circunferências dos MMSS, registradas nos questionários impressos.

As pacientes foram abordadas novamente em um segundo momento, Tempo 2 (T2), após aproximadamente 45 dias da cirurgia (com mais ou menos três dias devido aos finais de semana e feriados) em consulta pós-operatória ambulatorial, para responderem novamente os instrumentos de QVRS e realizar a perimetria dos MMSS.

### Análise dos resultados e estatística

Os dados foram codificados e inseridos em planilha eletrônica no Excel e validados após dupla entrada. Para a análise estatística foi utilizado o software *Statistical Package for Social Science for Windows* (SPSS), versão 29.0.1.0.

As variáveis categóricas foram analisadas segundo estatística descritiva com distribuições de frequência absoluta e relativa, enquanto que, para os instrumentos de QVRS, foram calculados seus escores, e em seguida medidas de centralidade (média) e de variabilidade (desvio-padrão). Os dados foram testados quanto à distribuição e, em seguida, para avaliar as diferenças nos escores de QVRS destas mulheres antes e após a cirurgia, foi realizado o teste T pareado.

Para avaliar a influência de variáveis sociodemográficas e clínicas sobre a QVRS foi utilizado o teste t de *Student* para grupos independentes de cinco preditores dicotômicos para com os instrumentos de QVRS, a saber: faixa etária (< 60 e ≥ 60 anos), se teve filho (sim e não), nível de escolaridade (sem estudo até ensino médio e ensino superior até pós-graduação), cirurgia (mastectomia e outros tipos) e linfedema (presença ou ausência); e correlações de *Pearson* para a variável idade em anos completos e *Spearman* para escolaridade em diferentes níveis.

Também foi realizada a análise de regressão linear múltipla da influência simultânea dos cinco preditores (linfedema, ter filhos, tipo de cirurgia, escolaridade e idade) sobre o desfecho primário: o escore de Estado Geral de Saúde (EGS/QV). O estudo considerou um nível de significância estatística de 5%, ou seja, α=0,05.

## RESULTADOS

Participaram 90 mulheres com média de idade de 55 anos (DP=11,91) (mínima de 18 e máxima de 82 anos). Predominaram mulheres que se autodeclararam brancas (60,0%), casadas ou que mantinham união estável (47,8%), e que possuía filhos (86,7%). Houve uma semelhança entre as que estavam economicamente ativas e aquelas que eram donas de casa, aposentadas ou pensionistas. Quanto ao grau de instrução, a maioria possuía ensino fundamental, seguido de ensino médio e superior completo. Houve relato de duas mulheres que nunca estudaram e uma com pós-graduação concluída, conforme apresenta a Tabela 1.

**Tabela 1:** Caracterização sociodemográfica da amostra (N=90), Uberaba, Minas Gerais, Brasil, 2022-2023

Variáveis	Características	Total n (%)
Cor autorrelatada	Branca Mulata Negra Amarela	54 (60,0) 18 (20,0) 17 (18,9) 1 (1,1)
Estado civil	Casada/União Estável Solteira Divorciada Viúva	43 (47,8) 20 (22,2) 16 (17,8) 11 (12,2)
Possui Filhos	Sim Não	78 (86,7) 12 (13,3)
Profissão/ Ocupação	Economicamente ativa Do lar, donas de casa Aposentada/pensionista Não trabalha	44 (48,9) 24 (26,7) 20 (22,2) 2 (2,2)
Nível de escolaridade	Não estudou Ensino Fundamental Incompleto Ensino Fundamental Completo Ensino Médio Incompleto Ensino Médio Completo Ensino Superior Incompleto Ensino Superior Completo Pós-graduação Completa	2 (2,2) 25 (27,8) 22 (24,4) 6 (6,7) 19 (21,1) 1 (1,1) 14 (15,6) 1 (1,1)

A Tabela 2 evidencia que houve uma pequena diferença entre as mamas afetadas, prevalecendo a direita (50,0%). A maioria das mulheres informou não ter histórico de câncer de mama na família (56,7%), foi submetida a cirurgia conservadora (66,7%) e não realizou quimioterapia (51,1%) nem radioterapia (83,3%).

**Tabela 2:** Caracterização clínica e terapêutica da amostra (N=90), Uberaba, Minas Gerais, Brasil, 2022-2023

Variáveis	Características	Total n (%)
Mama afetada	Direita Esquerda Ambas	45 (50,0) 43 (47,8) 2 (2,2)
Histórico Familiar	Não Sim	51 (56,7) 38 (42,3)
Cirurgia realizada	Conservadoras Mastectomias	60 (66,7) 30 (33,3)
Realizou quimioterapia	Não Sim	46 (51,1) 43 (47,9)
Realizou radioterapia	Não Sim	75 (83,3) 14 (15,7)

O linfedema no membro ipsilateral à mama afetada ocorreu em 37 (41,1%) mulheres no pós-operatório, em pelo menos um dos locais mensurados. A maior incidência do linfedema se concentrou em 14 cm acima do cotovelo, conforme apresenta a Tabela 3. O grau de linfedema apresentado nas mulheres foi considerado moderado (3 a 5 cm de diferença).

**Tabela 3:** Distribuição do linfedema no membro ipsilateral à mama operada da amostra (N=90), Uberaba, Minas Gerais, Brasil, 2022-2023

Mensuração do membro ipsilateral à mama operada	Linfedema presente n (%)	Linfedema ausente n (%)
14 cm acima do cotovelo	19 (21,1)	71 (78,9)
7 cm acima do cotovelo	9 (10,0)	81 (90,0)
7cm abaixo do cotovelo	14 (15,6)	76 (84,4)
14 cm abaixo do cotovelo	5 (5,6)	85 (94,4)
21 cm abaixo do cotovelo	13 (14,4)	77 (85,6)

A Tabela 4 mostra os resultados do QLQ-C30 e do QLQ-BR23. O Estado Geral de Saúde e as funções física, desempenho de papel, cognitiva e social apresentaram uma pequena redução do pré ao pós-operatório, tendo sido estatisticamente significativo apenas para desempenho de papel (p< 0,05), enquanto a função emocional não sofreu alteração.

**Tabela 4:** Média, desvio padrão e teste T pareado para as escalas e itens dos instrumentos QLQ-C30 e QLQ-BR23 (N=90), Uberaba, Minas Gerais, Brasil, 2022-2023

Escalas e itens	Média (DP) (Pré-operatório)	Média (DP) (Pós-operatório)	Valor de *p*
Estado geral de saúde/Qualidade de vida	82,77 (19,18)	81,94 (19,99)	0,74
Função física	90,81 (11,97)	88,44 (15,21)	0,14
Desempenho de papel	89,81 (20,85)	84,07 (21,91)	0,04^*^
Função cognitiva	75,18 (23,60)	74,44 (29,58)	0,81
Função social Função emocional	88,14 (21,23) 75,18 (23,80)	84,62 (23,33) 75,55 (26,04)	0,21 0,88
Fadiga	12,46 (17,69)	15,67 (19,41)	0,13
Náuseas e vômitos	8,14 (18,39)	6,85 (15,37)	0,49
Dor	11,84 (16,42)	15,92 (22,47)	0,09
Dispneia	10,36 (20,98)	7,03 (20,89)	0,18
Insônia	46,66 (44,07)	33,33 (40,59)	0,00^*^
Perda de apetite	14,07 (25,95)	12,59 (24,77)	0,62
Constipação	9,99 (21,46)	4,44 (15,95)	0,02^*^
Diarreia	7,77 (21,80)	7,77 (19,38)	1,00
Dificuldade financeira Imagem corporal Função sexual Satisfação sexual Perspectivas futuras Efeitos colaterais relacionados ao tratamento Sintomas da mama Sintomas do braço Perturbação com perda do cabelo	19,62 (28,21) 88,23 (20,22) 9,99 (19,31) 10,73 (22,80) 59,25 (38,95) 23,82 (17,85) 11,47 (15,12) 15,98 (23,64) 20,74 (35,18)	18,88 (30,00) 86,46 (22,50) 14,81 (21,70) 14,81 (23,49) 71,47 (32,59) 23,50 (20,43) 15,55 (18,36) 23,01 (25,91) 12,59 (27,63)	0,82 0,42 0,08 0,21 0,00^*^ 0,87 0,10 0,02^*^ 0,03^*^

* *Diferença significativa em nível de 5% (p<0,05).*

Quanto aos sintomas, a principal queixa foi insônia que juntamente com a constipação tiveram uma redução estatisticamente significativa do pré ao pós-operatório, enquanto houve um aumento na fadiga e na dor no pós-operatório.

Em relação a avaliação do QLQ-BR23, as mulheres referiram uma diminuição da imagem corporal e um aumento nos sintomas da mama e do braço, sendo estatisticamente significativo para este último. Apesar deste aumento nas queixas, também relataram uma melhora na função e satisfação sexual, nas perspectivas futuras e em uma redução na perturbação com a perda do cabelo, estatisticamente significativo para estas duas últimas, conforme mostra Tabela 4.

Na análise dos preditores sobre a QVRS, observaram-se diferenças estatisticamente significantes para o sintoma insônia (p=0,04), no qual as mulheres que possuíam filhos (78-70,2%) relataram apresentar mais insônia (M=36,75, DP= 41,11) do que as que não tinham filhos (M=11,11, DP= 29,58) e as mulheres que foram submetidas a mastectomia (30-27,0%) apresentaram melhor EGS/QV (p=0,01, M=89,44, DP=12,56) e perspectivas futuras (PF) (p=0,04, M=81,10, DP= 20,87) do que as que realizaram outros tipos de cirurgia (EGS/QV: M=78,19, DP= 21,97; PF: M=66,66, DP=36,30).

Com relação à influência simultânea dos cinco preditores (linfedema, ter filhos, tipo de cirurgia, escolaridade e idade) sobre o desfecho primário (escore de Estado Geral de Saúde), o modelo de regressão linear mútipla apontou que o tipo de cirurgia realizada foi a única variável que explicou maiores preditores de EGS nestas mulheres (p = 0,01), conforme Tabela 5.

**Tabela 5:** Teste de regressão linear múltipla dos preditores com o Estado Geral de Saúde/Qualidade de vida no pós-operatório (N = 90), Uberaba, Minas Gerais, Brasil, 2022-2023

Estado Geral de Saúde	β	Valor de *p*
Linfedema	-0,06	0,55
Filhos	-0,09	0,41
Cirurgia	0,26	0,01^*^
Escolaridade	-0,01	0,90
Idade	0,09	0,42

* *Diferença significativa em nível de 5% (p<0,05).*

Aplicou-se a correlação de *Pearson* para a variável idade em anos completos (p=0,42) e a correlação de *Spearman* para a variável escolaridade em diferentes níveis (p=0,90), contudo, nenhuma apresentou resultado estatisticamente significante.

## DISCUSSÃO

O perfil das mulheres foi compatível com estudo desenvolvido em Franca-SP, que encontrou média de idade de 59,9 anos e a maioria autodeclaradas brancas (78,1%). Contudo, foi discrepante no tipo de cirurgia, predominando as mastectomias em 55,6% das pacientes^(16)^. Já em estudo desenvolvido na Nigéria, a média de idade das mulheres foi menor, de 45,94 anos (DP=9,08), a maioria era casada (58,3%) e tinha de um a quatro filhos, e também divergiu com relação às cirurgias, sobrepujando a mastectomia (83,3%) sobre a tumorectomia (16,7%)^(17)^.

Quanto ao linfedema, na presente investigação, o mesmo ocorreu em 41,1% das mulheres. Em uma pesquisa desenvolvida na capital paulista, o linfedema foi detectado em 47,5% das mulheres, dentre estas, 57,1% foram submetidas a cirurgias conservadoras e 42,9% às radicais, e ainda encontraram uma associação estatisticamente significativa (p=0,06) com o tempo pós-operatório, aumentando a chance da ocorrência do linfedema após seis meses de cirurgia^(7)^. Um aumento na taxa de linfedema em 10% e 18% também foi encontrado após 12 e 18 meses de pós-operatório, respectivamente, nos Estados Unidos^(18)^.

Com relação a QVRS, observou-se pequeno declínio nas funções avaliadas do pré ao pós-operatório, corroborando o estudo desenvolvido nos Estados Unidos, no qual os domínios físico, psicológico e social reduziram no primeiro mês pós-cirurgia mas, entretanto, foram melhorando cerca de seis meses após a cirurgia^(18)^. Na Arábia Saudita, os desafios psicológicos, sociais e/ou emocionais impactaram significativamente no bem-estar geral e na QVRS^(19)^. Enquanto na Nigéria o sofrimento psicológico foi significativamente associado à QV (p=0,001), visto que as mulheres que referiram sofrimento psicológico tiveram menor QV (M=68,42, DP=16,22) do que aquelas sem sofrimento (M=85,52, DP=15,99)^(17)^.

Dentre os sintomas, destaca-se a insônia, que mesmo tendo sofrido uma redução no pós-operatório, foi a mais referida nos dois momentos avaliados e estatisticamente mais relatada pelas mulheres com filhos. A insônia é considerada um sintoma comum e está relacionada a vários fatores aos quais a mulher é exposta durante o tratamento da doença, como dor e desconforto local, efeitos colaterais do tratamento, e aflição emocional^(20)^.

A presente investigação encontrou um relato da diminuição de imagem corporal, que apesar de não ter sido estatisticamente significativo, corrobora uma revisão integrativa cujo estudo demonstrou que as mulheres mastectomizadas passam por grande desafio devido à retirada da mama e tem sua autopercepção e autoestima afetadas, comprometendo sua sexualidade^(21)^.

A cirurgia na mama provocou conflitos nos relacionamentos afetivos em mulheres mais jovens (entre 18 e 40 anos) pois, ao retornarem à prática sexual, muitas apresentavam vergonha de ficarem nuas perante os cônjuges e exibirem um “corpo defeituoso”, fazendo lembrar que estavam doentes^(22)^.

A mudança da aparência física ocasionada pela mastectomia e perda do cabelo causaram déficit na imagem corporal destas mulheres jovens. Elas referiram que a prática sexual foi modificada após o diagnóstico e tratamento, e o retorno à mesma só foi possível com o apoio social e afetivo, principalmente do companheiro^(22)^.

Neste estudo, os escores da função emocional não sofreram alteração, do pré ao pós-operatório. Contudo, estudo revela que ser mais jovem (p=0,005) e ter encaminhamento para tratamento de linfedema (p=0,006) foram associados a pior QVRS no âmbito psicológico^(18)^.

Estudo libanês que utilizou os mesmos instrumentos de QVRS na avaliação pré e um dia após a cirurgia conservadora da mama encontrou redução estatística e clinicamente significativa para imagem corporal e para as funções física e emocional no pós-operatório, além de uma mudança positiva no funcionamento físico entre mulheres casadas. A imagem corporal e perspectivas futuras reduzidas foram elencadas como fatores de risco para uma má qualidade de vida^(23)^, colaborando com a presente investigação cujas mulheres que tiveram cirurgias conservadoras apresentaram escores menores para perspectivas futuras e qualidade de vida do que as que realizaram mastectomia.

Entretanto, os resultados divergem de outra pesquisa que também comparou com a opção de cirurgia de reconstrução mamária, no qual evidenciou-se que as mulheres submetidas à cirurgias oncoplásticas apresentaram melhor QV do que as que realizaram cirurgias conservadoras, e estas tiveram melhor QV do que aquelas que realizaram a mastectomia sem reconstrução^(24)^.

Em mulheres egípcias que realizaram mastectomia, a QV foi influenciada por vários fatores: elas obtiveram uma QV global de 42,0 e as escalas mais prejudicadas foram de desempenho de papel e a função emocional, já os sintomas mais presentes foram fadiga (65%), insônia (61%) e dor (60%)^(25)^.

Diante das várias mudanças que podem ocorrer com a realização de cirurgias para tratamento do câncer de mama, os profissionais de saúde devem propor intervenções que possam ajudar estas mulheres na redução dos sintomas físicos e psicossociais, e na melhoria das funções e QVRS.

Apesar de a presente investigação não ter encontrado associação estatisticamente significativa de linfedema com a qualidade de vida, esta complicação pós-operatória também deve ser avaliada e tratada. Em estudo que utilizou a terapia complexa descongestiva (TCD) adaptada em uma paciente para o controle da sintomatologia e do volume do linfedema, os fisioterapeutas realizaram orientações de cuidados com a pele, exercícios passivos domiciliares e enfaixamento compressivo duas vezes por semana e, apesar de não ter sido realizada drenagem linfática, houve redução do volume do linfedema. Na etapa seguinte, visando a manutenção, utilizaram uma braçadeira compressiva diariamente. O tratamento também proporcionou redução da dor e da sensação de peso do membro, relatada pela paciente, cuja adesão ao tratamento proposto influenciou nos resultados positivos^(26)^.

Visando a melhora de sintomas, inclusive da insônia, sugere-se realização de terapias psicossociais^(17)^ e comportamentais, tais como terapia cognitivo-comportamental, ioga, práticas mente-corpo^(20)^, cinesioterapia^(27)^, além de orientações quanto a prática de atividades físicas, alimentação equilibrada, manutenção de hábitos de vida saudáveis^(28)^ e realização de higiene do sono, bem como ofertar suporte social, como prestação de assistência emocional, informativa e instrumental às mulheres que possuem filhos dada pelos profissionais de saúde que assistem essa população. Tanto enfermeiros quanto outros profissionais como fisioterapeuta, psicólogo e educador físico devem propor ações que visem a melhora da QVRS das mulheres durante o tratamento para o câncer de mama.

Um exemplo de ação é a realização de atividade física, considerada segura e pode ser praticada durante todo o tratamento, desde que a mulher seja avaliada e acompanhada, inclusive no pós-operatório, resultando na redução de sintomas psicológicos, como ansiedade e depressão, e outros como dor, fadiga, distúrbios do sono, náuseas e vômitos, além de melhorar as funcionalidades e consequentemente o bem-estar e QVRS^(28)^.

Outra intervenção usada para melhorar a qualidade de vida é a cinesioterapia. Aplicação no pós-operatório de câncer de mama, cujo protocolo incluiu 10 sessões de alongamentos, exercícios ativos-livres e exercícios resistidos aplicados por fisioterapeuta, demonstrou que as pacientes que realizaram a terapia apresentaram melhora significativa na função física (p= 0,01), desempenho de papel (p= 0,02), fadiga (p= 0,03), dor (p= 0,01), insônia (p = 0,02, sintomas da mama (p= 0,01) e do braço (p= 0,01) e piora da diarreia (p= 0,02). Os outros escores avaliados pelos instrumentos QLQ-C30 e QLQ-BR-23 também apresentaram melhoras, contudo sem diferença significativa^(27)^.

### Limitações do estudo

Como limitações destaca-se a dificuldade em contatar as mulheres para coleta de dados no pós-operatório devido a antecipação ou reagendamento de consultas. Acrescenta-se ainda a limitação da não realização de novas avaliações ao longo do tempo. Esta última, embora seja considerada limitação, mostra-se como possibilidade de realização de novos estudos com a mesma população, com desenhos diferentes.

### Contribuições para a área da Enfermagem, Saúde ou Políticas Públicas

O estudo contribui com a equipe de saúde que assiste essa população, pois fornece informações de possíveis acometimentos ocorridos no pré e pós-operatório destas mulheres e, com isso, tanto profissionais de enfermagem quanto de fisioterapia e outras categorias podem propor intervenções para amenizar tais sintomas e disfunções que podem acometer a QVRS das pacientes.

## CONCLUSÕES

A presença de linfedema ocorreu em menos da metade das mulheres avaliadas. Do pré ao pós-operatório, houve mudanças estatisticamente significativas (p<0,05) negativamente com a redução no desempenho de papel e aumento nos sintomas do braço, e positivamente com redução da insônia, constipação, e na perturbação com a perda do cabelo e aumento nas perspectivas futuras.

A insônia foi o sintoma mais prevalente em ambos os momentos. Como preditores, encontrou-se nas associações diferenças estatisticamente significativas, indicando que as mulheres que possuem filhos sofreram com mais insônia dos que as que não possuem, e as mulheres mastectomizadas apresentaram melhor EGS/QV e perspectivas futuras do que as que realizaram cirurgia conservadora.

Desse modo, esta pesquisa ressalta a importância de os profissionais de saúde avaliarem o surgimento de linfedema e de outros fatores predisponentes às mudanças na qualidade de vida de mulheres com câncer de mama submetidas a cirurgias, e proporem intervenções que possam amenizar ou prevenir sintomas decorrentes do tratamento, aumentar as funções biopsicossociais e, consequentemente, proporcionar um atendimento integral e melhor QVRS a estas mulheres.

## Data Availability

Os dados de pesquisa estão disponíveis no corpo do artigo.
